# Dental Caries and Autism: An Assessment of Prevalence and Risk Factors in Children With Autism Spectrum Disorder in Arar, Saudi Arabia

**DOI:** 10.7759/cureus.92154

**Published:** 2025-09-12

**Authors:** Ahmed AlSumur, Mustafa A Ahubail, Hassan A Alali, Abdulrahman N Alanazi, Wael H Alanazi, Fahad I Alanazi, Nahid A Mohammed, Saud Albakr, Nahi Sabih Q Alruwaili

**Affiliations:** 1 Department of Dentistry, Al Nairyah Hospital, Al Nairyah, SAU; 2 Department of Dentistry, Aljafer Primary Health Center, Al Ahsa, SAU; 3 Department of Nursing, Eradah Complex for Mental Health, Arar, SAU; 4 Department of Mental Health, Eradah Complex for Mental Health, Arar, SAU; 5 Department of Dentistry, Riyadh Second Health Cluster, Riyadh, SAU; 6 Department of Psychiatry, Eradah Complex for Mental Health, Arar, SAU

**Keywords:** autism spectrum disorder, children, dental caries, oral health, oral hygiene, prevalence, saudi arabia

## Abstract

Background: Autism spectrum disorder (ASD) is a neurodevelopmental condition characterized by challenges in social interaction, communication, and repetitive behaviors, which significantly impact oral health due to difficulties in maintaining oral hygiene. Understanding the prevalence of dental caries in children with ASD can guide healthcare providers in implementing better oral care practices and preventive strategies. This study investigates the prevalence of dental caries among children with ASD in Arar, Saudi Arabia.

Methods: This cross-sectional study was conducted at the Maternal and Children Hospital in Arar, Saudi Arabia. The study included 44 children aged 4 to 17 years diagnosed with ASD by pediatricians or psychologists. A self-administered questionnaire collected data on demographics and oral hygiene behaviors, followed by oral examinations adhering to WHO criteria using the DMFT (Decayed, Missing, Filled Teeth) and deft (decayed, extracted, filled, teeth) indices. Data were analyzed using IBM SPSS Statistics for Windows, version 26 (IBM Corp., Armonk, NY, USA), with statistical tests including chi-square, Mann-Whitney U test, and binary logistic regression.

Results: Among the 44 participants, 81.8% were male subjects and 18.2% were female subjects. Most of the sample consists of younger children in the primary dentition stage (54.5%), followed by those in the mixed dentition stage (29.5%). The prevalence of dental caries was 38.6%. Age was significantly associated with caries prevalence (p = 0.022), whereas gender, number of siblings, birth order, level of education, teeth brushing frequency, flossing frequency, type of primary health institution, frequency of dental visits, parents' education, and family income showed no significant association. The mean DMFT and deft scores were 0.64 ± 1.33 and 0.91 ± 1.78, respectively.

Conclusion: The study revealed a high prevalence of dental caries among children with ASD in Arar, Saudi Arabia, with age being a significant factor. These findings highlight the need for targeted oral hygiene practices and regular dental visits for children with ASD to manage and prevent dental caries effectively. Further research is essential to develop preventive strategies and improve access to dental care for this vulnerable population.

## Introduction

Autism spectrum disorder (ASD) is a multifaceted neurodevelopmental condition marked by ongoing difficulties in social interactions, communication, and restricted or repetitive behaviors [1]. The term “spectrum” is used to describe the broad range of symptoms and varying levels of severity associated with ASD. Typically, ASD appears in early childhood and persists throughout life, though symptoms may evolve over time. While the exact cause of ASD is unknown, it is thought to result from a combination of genetic and environmental factors.

Autism is more commonly diagnosed in boys, with a four times higher likelihood compared to girls [2]. The disorder is observed with varying severity across all races and groups worldwide, and there has been a reported increase in its prevalence globally. For example, in Saudi Arabia, many studies have examined the prevalence of ASD among the Saudi population. Their data has shown a considerable increase in children diagnosed with ASD when comparing past and present studies. In addition, one of the most recent studies investigating the prevalence of ASD in the Saudi Arabian population reported an estimated rate of 1 in 40, or 25 per 1000 individuals. The study also found that male individuals are at least three times more likely than female individuals to be diagnosed with ASD [3-5]. Autism significantly impacts oral health due to challenges with motor coordination, sensory sensitivities, and difficulty maintaining consistent oral hygiene habits [6].

Dental caries is common among children with ASD. The occurrence of dental caries in children with ASD can be similar [7,8] to or even lower than [9] that of the general population. However, some studies have found a higher prevalence of dental caries in children with ASD compared to the general population [10]. A recent systematic review and meta-analysis study confirmed that the prevalence of dental caries among children and young adults with ASD is 60.6% [11]. In some children with ASD, sweet foods are frequently used as behavioral rewards [12,13]. This practice can contribute to the development of dental caries, as the consumption of foods high in sugar, along with their prolonged intake, plays a significant role in caries development [14]. Dental caries occurs when the enamel and dentin of teeth are demineralized by organic acids. These acids are produced by bacteria in dental plaque through the anaerobic metabolism of dietary sugars. Dental caries is a preventable disease, and epidemiological studies have shown that behavior plays a significant role in its development.

Poor oral hygiene practices, such as inadequate toothbrushing, are key factors contributing to caries development in children [15]. Evidence suggests that brushing teeth properly twice a day is sufficient to maintain oral health [16]. The American Academy of Pediatrics recommends supervising toothbrushing until around 8 years of age, while the New Zealand Ministry of Health recommends supervision until around 6 years of age [12,13]. This is because children under 6 often lack the skills to effectively remove plaque on their own, increasing their risk of developing dental caries. However, proper oral hygiene practices can be challenging for many children with ASD. Children with ASD often have physical impairments that make it difficult for them to perform self-oral hygiene practices, including toothbrushing, without assistance. Therefore, rinsing after meals supports oral hygiene by compensating for common challenges such as sensory sensitivities, aversions to toothbrushing, and restricted diets high in carbohydrates or sugars. Many children with ASD may have difficulty with fine motor skills, making thorough brushing less effective. Rinsing with water after eating helps remove residual food and sugars, reduces bacterial growth, and lowers caries risk between brushing sessions. They may require extensive training or assistance from their parents or caregivers to establish these practices as daily routines. For parents and caregivers, helping children with ASD adopt proper oral hygiene practices can be stressful due to the time and energy required [12].

Regular visits are crucial in treating dental caries and preventing further damage due to untreated dental caries [17]. However, factors such as the child's challenging behavior and difficulties in finding a dentist with the necessary skills or willingness to work with individuals with disabilities can influence dental visit behaviors among children with ASD [12]. Despite the significant risk of dental caries in children with ASD, there remains limited literature on dental caries and oral health behaviors in this population, especially in developing countries. The aim of this study is to assess the prevalence and identify the risk factors of dental caries in children with ASD.

## Materials and methods

Study design and settings

This cross-sectional study was conducted at the Maternal and Children Hospital in Arar, Saudi Arabia. The study aimed to investigate the prevalence of dental caries among children diagnosed with ASD. Written consent was obtained from the legal guardians of all participants before conducting any dental examinations. The ethical approval was obtained from the Local Committee for Research Ethics, Northern Borders Health, Ministry of Health, Kingdom of Saudi Arabia (Approval No: NB-IRB-023-03-025, dated May 18, 2023).

Study population

Participants were selected from the regular attendees of the behavioral disturbance clinic at the Maternal and Children Hospital. They were diagnosed with ASD by pediatricians, medical specialists, or psychologists. The study included 44 children aged between 3 to 17 years, as this age range typically covers the pediatric patient population. Participants who did not meet the diagnostic criteria for ASD, exhibited uncooperative behavior during the study, fell outside the specified age range (under 3 years old or over 17 years old), or whose legal guardians did not provide written consent were excluded from the study. Additionally, cases with incomplete or inappropriate responses in the questionnaire form were not included in the final analysis.

Data collection tool and variables

A self-administered hardcopy questionnaire (see Appendices) was distributed to the parents/guardians of the participant before the oral examination. Initially, an explanatory section outlined the study's objectives, potential benefits, and risks of participation. Parents/guardians were given the option to consent or decline participation, with the assurance that they could withdraw their child from the study at any time by signing the consent form.

The questionnaire included demographic information such as age, gender, nationality, number of siblings, and birth order among siblings. Participants' educational levels (ranging from nursery to secondary school or non-formal education) were also queried. The questionnaire primarily consisted of closed-ended questions. Most variables (e.g., gender, age category, teeth brushing frequency, flossing frequency, last dental visit, parental education) were captured using categorical or numerical responses with predefined options. Also, parents/guardians were specifically asked to confirm their child's ASD diagnosis.

For the following questions, in the second part of the questionnaire (Oral Hygiene Behaviors), parents/guardians were asked to report their child’s oral hygiene behaviors, including the frequency of teeth brushing, flossing, and the number of visits to a dental clinic in the past six months in either governmental or private sectors. Additionally, parents/guardians provided information on their own educational attainment and family socioeconomic status, categorized into four income classes: Class 1 (10,000 SAR or less per month), Class 2 (between 10,000 and 20,000 SAR per month), Class 3 (between 20,000 and 40,000 SAR per month), and Class 4 (more than 40,000 SAR per month) [18]. Each participant was assigned a code to maintain anonymity while answering the questionnaire, ensuring confidentiality and privacy protection throughout the research process. Convergent validity was assessed by examining correlations between oral hygiene behaviors and clinical caries indices.

Oral examination

Diagnostic criteria for dental caries adhered to the World Health Organization (WHO) recommendations, utilizing the DMFT (Decayed, Missing, Filled Teeth) and deft (decayed, extracted, filled, teeth) indices [19]. Teeth with cavitated lesions or those where cavity status was uncertain were categorized as Decayed Teeth (DT). Missing teeth due to caries extraction were recorded as Missing Teeth (MT), while teeth with permanent restorations were classified as Filled Teeth (FT), applicable to both primary and permanent dentitions. The examiners conducting the oral examinations were trained dental professionals, specifically dentists who followed standardized protocols consistent with WHO recommendations. They utilized appropriate dental instruments such as tongue depressors, mouth mirrors, and headlights to ensure accurate and reliable assessment of dental caries using the DMFT and deft indices. All examiners were calibrated prior to data collection to maintain consistency and reliability in diagnosing caries and recording findings [19,20]. Following the oral evaluation, examiners provided parents/guardians with a summary of their child's oral health condition and offered guidance on appropriate next steps for dental care and hygiene education.

Statistical analysis

All the analyses and calculations were performed using IBM SPSS Statistics for Windows, version 26 (IBM Corp., Armonk, NY, USA). The normality of the continuous variables was checked using the Kolmogorov-Smirnov test. The data was presented as mean ± SD or median (IQR) for continuous variables, e.g., age, number of siblings, DMFT, and deft, and as proportions for categorical variables, e.g., gender, birth order, level of education, diagnosis of autism, teeth brushing frequency, flossing frequency, near primary health institution, last visit in the previous six months, fathers’ education, and mothers’ education. Caries was considered positive when (DMFT/deft > 0). The Mann-Whitney U test was performed to compare the significant median differences for (DMFT/deft) scores between genders. A chi-square test was used to compare categorical variables. A Mann-Whitney U test was used to determine the association between caries and continuous demographic data. A binary logistic regression analysis was performed to identify the relationship between caries and associated risk factors. The odds ratio (OR) and confidence interval (95% CI) were reported. A p-value less than 0.05 was considered statistically significant. Internal consistency of the questionnaire was assessed using Cronbach’s alpha.

## Results

**Figure 1:**
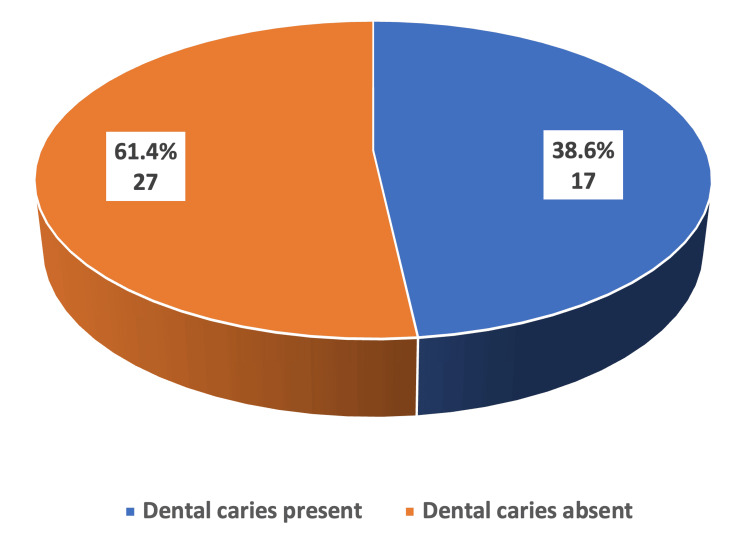
Prevalence of caries among the included autistic children (N=44)

Out of 1000 children who visited the behavior disturbance clinic in the Maternal and Children Hospital in Arar city, Northern Borders province, Saudi Arabia, 44 children were included in the study as they had been diagnosed with ASD by a consultant psychiatrist. The majority of the sample consists of younger children in the primary dentition stage (54.5%), followed by those in the mixed dentition stage (29.5%). The percentage of male participants was higher (81.8%) than that of female participants (18.2%). Most autistic children 31.8% had a primary school education. Among autistic children, 40.9% brush their teeth once a day. Additionally, 68.2% and 65.9% of the mothers and fathers were graduates, respectively. 38.6% of the autistic children presented with caries (DMFT > 0). The median (Q1-Q3) caries score DMFT and deft for the entire study population were 0(0-0) and 0(0-1), respectively (Table 1, Figure 1).

**Table 1:** Baseline and clinical characteristics of the included patients No children were born as the 8th birth order in the study population. IQR: interquartile range; DMFT: decayed, missing, filled teeth; deft: decayed, extracted, filled, teeth

Variable	Total (n=44) Median (Q1-Q3)/n (%)
Age (years)	
Primary dentition (3-6)	24 (54.5)
Mixed dentition (7-11)	13 (29.5)
Permanent dentition (12-17)	7 (15.9)
Gender	
Male	36 (81.8)
Female	8 (18.2)
Family siblings Median (IQR)	4 (2-6)
Birth order	
1	13 (29.5)
2	10 (22.7)
3	5 (11.4)
4	6 (13.6)
5	3 (6.8)
6	4 (9.1)
7	1 (2.3)
9	1 (2.3)
10	1 (2.3)
Level of education	
Nursery	5 (11.4)
Kindergarten	3 (6.8)
Primary school	14 (31.8)
Intermediate	2 (4.5)
None	20 (45.5)
Teeth brushing frequency	
One time per day	18 (40.9)
Two times per day	3 (6.8)
Three times or more per day	2 (4.5)
None per day	21 (47.7)
Flossing frequency	
One time per day	1 (2.3)
None per day	43 (97.7)
Nearest primary health institution	
Government	43 (97.7)
Private	1 (2.3)
Last visit to the dental clinic during the last 6 months	
Once	12 (27.3)
Twice	1 (2.3)
More than 3 times	1 (2.3)
None	30 (68.2)
Fathers’ education	
Primary school	2 (4.5)
Intermediate school	1 (2.3)
Secondary school	9 (20.5)
Graduates	29 (65.9)
None	3 (6.8)
Mothers’ education	
Primary school	2 (4.5)
Intermediate school	2 (4.5)
Secondary school	10 (22.7)
Graduates	30 (68.2)
Family income	
≤10000	23 (52.3)
10000-20000	19 (43.2)
21000-40000	2 (4.5)
DMFT Median (Q1-Q3)	0 (0-0)
deft Median (Q1-Q3)	0 (0-1)
Caries	
Yes	17 (38.6)
No	27 (61.4)

The questionnaire demonstrated excellent internal consistency, indicated by a high Cronbach’s alpha value of 0.981. This suggests that the four items included in the scale are highly correlated and measure the same underlying construct reliably. Such a strong alpha value reflects the consistency of responses across these items, supporting the reliability of the questionnaire for the study (Table 2).

**Table 2:** Reliability statistics

Cronbach's alpha	Number of Items
0.981	4

Table 3 shows, that the median score for DMFT was the same among male participants with 0 IQR (0-0) and female participants with 0 (0-1). Similarly for deft score, the median scores were similar for gender between both male participants with median = 0 IQR (0, 2) and female participants with median = 0 (0, 1). Hence, there was no statistically significant difference in both DMFT and deft scores between male and female participants, as p-values of 0.848 and 0.593 >0.05.

**Table 3:** Comparison of DMFT/deft scores by gender DMFT: decayed, missing, filled teeth; deft: decayed, extracted, filled, teeth

Variables	Gender		p-value
	Male	Female	
	Median (Q1, Q3)	Median (Q1, Q3)	
DMFT	0 (0,0)	0 (0,1)	0.848
deft score	0 (0,2)	0 (0,1)	0.593

In a comparison of caries prevalence among autistic children with various baseline and clinical characteristics, there was a significant association between age and the prevalence of caries (p = 0.022). Caries were more common in the mixed dentition group (41.2%), followed by the permanent dentition group (29.4%), and the primary dentition group (29.4%). However, the highest proportion of children without caries was found in the primary dentition group (70.4%). No significant differences were found in caries prevalence based on gender among autistic children (p = 0.942), a number of siblings (p = 0.643), birth order (p = 0.073), level of education (p = 0.246), teeth brushing frequency (p = 0.095), flossing frequency (p = 0.422), type of primary health institution (p = 0.422), frequency of dental visits (p = 0.358), fathers' education (p = 0.149), mothers' education (p = 0.302), or family income (p = 0.167). This suggests that age is the most significant factor associated with caries prevalence among autistic children, while other demographic and socio-economic factors do not show a significant impact within this population.

Teeth brushing frequency showed a significant negative correlation with DMFT scores (r = -0.382, p = 0.010), indicating that higher brushing frequency is associated with lower caries experience, supporting the validity of brushing frequency as a measure of oral hygiene behavior. Additionally, DMFT and dmft scores were strongly positively correlated (r = 0.486, p < 0.001), confirming these indices as consistent measures of caries burden. Flossing frequency was not significantly correlated with other variables, possibly due to the low prevalence of flossing in the study population (Table 4).

**Table 4:** Comparison of caries presence against sociodemographic characteristics of autistic children No children were born as the 8th birth order in the study population. IQR: interquartile range

Variable	Caries among autistic children		p-value
	Yes n (%)	No n (%)	
Age (Years)			
Primary dentition (3-6)	5 (29.4)	19 (70.4)	0.022
Mixed dentition (7-11)	7 (41.2)	6 (22.2)	
Permanent dentition (12-17)	5 (29.4)	2 (7.4)	
Gender			
Male	14 (82.4)	22 (81.5)	0.942
Female	3 (17.6)	5 (18.5)	
Family siblings Median (IQR)	4.00 (2.00-6.00)	4.00 (2.00-5.00)	0.643
Birth order			
1	7 (41.2)	6 (22.2)	0.122
2	3 (17.6)	7 (25.9)	
3	0 (0.0)	5 (18.5)	
4	2 (11.8)	4 (14.8)	
5	0 (0.0)	3 (11.1)	
6	3 (17.6)	1 (3.7)	
7	0 (0.0)	1 (3.7)	
9	1 (5.9)	0 (0.0)	
10	1 (5.9)	0 (0.0)	
Level of education			
Nursery	0 (0.0)	5 (18.5)	0.246
Kindergarten	1 (5.9)	2 (7.4)	
Primary school	8 (47.1)	6 (22.2)	
Intermediate	1 (5.9)	1 (3.7)	
None	7 (41.2)	13 (48.1)	
Teeth brushing frequency			
One time per day	8 (47.1)	10 (37.0)	0.095
Two times per day	2 (11.8)	1 (3.7)	
Three times or more per day	2 (11.8)	0 (0.0)	
None per day	5 (29.4)	16 (59.3)	
Flossing frequency			
One time per day	0 (0.0)	1 (3.7)	0.422
None per day	17 (100)	26 (96.3)	
Nearest primary health institution			
Government	17 (100)	26 (96.3)	0.422
Private	0 (0.0)	1 (3.7)	
Last visit to the dental clinic in the previous 6 months			
Once	6 (35.3)	6 (22.2)	0.358
Twice	1 (5.9)	0 (0.0)	
More than 3 times	0 (0.0)	1 (3.7)	
None	10 (58.8)	20 (74.1)	
Fathers’ education			
Primary school	0 (0.0)	2 (7.4)	0.149
Intermediate school	0 (0.0)	1 (3.7)	
Secondary	3 (17.6)	6 (22.2)	
Graduates	11 (64.7)	18 (66.7)	
None	3 (17.6)	0 (0.0)	
Mothers’ education			
Primary school	1 (5.9)	1 (3.7)	0.302
Intermediate school	2 (11.8)	0 (0.0)	
Secondary	3 (17.6)	7 (25.9)	
Graduates	11 (64.7)	19 (70.4)	
Family income			
≤10000	9 (52.9)	14 (51.8)	0.167
10000-20000	6 (35.3)	13 (48.1)	
21000-40000	2 (11.8)	0 (0.0)	

The demographic and clinical characteristics of the study participants, stratified by gender, show minimal differences across most variables. The age distribution indicates that the majority of both male (58.3%) and female (37.5%) participants are in the primary dentition group, with no significant difference between the two (p=0.582). The prevalence of caries was similar between male (38.9%) and female (37.5%) (p=0.942) participants. The number of siblings and birth order also did not show significant variation by gender, nor did the participants' levels of education, teeth brushing, and flossing frequency. In terms of parental education, a higher proportion of fathers and mothers of female participants were graduates (100%) compared to males (58.3% for fathers, 61.1% for mothers), though this difference was not statistically significant. The income levels also appeared relatively similar between genders, with most participants coming from families earning ≤10,000 (52.8% males, 50% females). This indicates that gender does not significantly influence the baseline and clinical characteristics of autistic children in this study (Table 5).

**Table 5:** Comparison of gender against sociodemographic characteristics No children were born as the 8th birth order in the study population

Variable	Gender		p-value
	Male n (%)	Female n (%)	
Age (Years)			
Primary dentition (3-6)	21 (58.3)	3 (37.5)	0.582
Mixed dentition (7-11)	10 (27.8)	3 (37.5)	
Permanent dentition (12-17)	5 (13.9)	2 (25.0)	
Caries			
Yes	14 (38.9)	3 (37.5)	1.000
No	22 (61.1)	5 (62.5)	
Family siblings Median (IQR)	4.00 (2.00-6.00)	3.00 (2.25-5.50)	0.734
Birth order			
1	8 (22.2)	5 (62.5)	0.615
2	9 (25.0)	1 (12.5)	
3	4 (11.1)	1 (12.5)	
4	5 (13.9)	1 (12.5)	
5	3 (8.3)	0 (0.0)	
6	4 (11.1)	0 (0.0)	
7	1 (2.8)	0 (0.0)	
9	1 (2.8)	0 (0.0)	
10	1 (2.8)	0 (0.0)	
Level of education			
Nursery	4 (11.1)	1 (12.5)	0.412
Kindergarten	3 (8.3)	0 (0.0)	
Primary school	10 (27.8)	4 (50.0)	
Intermediate	1 (2.8)	1 (12.5)	
None	18 (50.0)	2 (25.0)	
Teeth brushing frequency			
One time per day	14 (38.9)	4 (50.0)	0.231
two times per day	2 (5.6)	1 (12.5)	
Three times or more per day	1 (2.8)	1 (12.5)	
None per day	19 (52.8)	2 (25.0)	
Flossing frequency			
One time per day	1 (2.8)	0 (0.0)	0.633
None per day	35 (97.2)	8 (100.0)	
Nearest primary health institution			
Government	35 (97.2)	8 (100.0)	1.000
Private	1 (2.8)	0 (0.0)	
Last visit to the dental clinic in the previous 6 months			
Once	9 (25.0)	3 (37.5)	0.834
Twice	1 (2.8)	0 (0.0)	
More than 3 times	1 (2.8)	0 (0.0)	
None	25 (69.4)	5 (62.5)	
Fathers’ education			
Primary school	2 (5.6)	0 (0.0)	0.281
Intermediate school	1 (2.8)	0 (0.0)	
Secondary	9 (25.0)	0 (0.0)	
Graduates	21 (58.3)	8 (100.0)	
None	3 (8.3)	0 (0.0)	
Mothers’ education			
Primary school	2 (5.6)	0 (0.0)	0.207
Intermediate school	2 (5.6)	0 (0.0)	
Secondary	10 (27.8)	0 (0.0)	
Graduates	22 (61.1)	8 (100.0)	
Family income			
≤10000	19 (52.8)	4 (50.0)	0.086
10000-20000	16 (44.4)	3 (37.5)	
21000-40000	1 (2.8)	1 (12.5)	

The table presents the results of a logistic regression analysis investigating the sociodemographic factors affecting caries prevalence. None of the sociodemographic variables (age, gender, nationality, level of education, flossing frequency, nearest primary health institution, last visit to a dental clinic in the previous six months, family siblings, birth order, teeth brushing frequency, fathers education, mothers education, and family income) had a p-value < 0.05 indicating that the model does not provide any significant predictors for caries (Table 6).

**Table 6:** Logistic regression analysis on patients’ socio-demographic characteristics and caries OR: odds ratio; CI: confidence interval; NA: not applicable

Variables	OR	(95% CI)		p-value
Age (Years) (Permanent dentition)				
Primary dentition	4.360E+59	0.000	0.000	1.000
Mixed dentition	7.308E+44	0.000	0.000	1.000
Gender (Female)				
Male	2.976E+15	0.184	136.664	0.997
Family siblings	2927312.820	0.000	0.000	0.999
Birth order (10)				
1	0.000	0.000	0.000	1.000
2	0.000	0.000	0.000	1.000
3	0.000	0.000	0.000	1.000
4	0.000	0.000	0.000	1.000
5	0.000	0.000	0.000	0.999
6	0.000	0.000	0.000	1.000
7	0.000	0.000	0.000	0.999
8	0.000	0.000	0.000	1.000
9	0.000	NA	NA	NA
Level of education (None)				
Nursery	0.000	0.000	0.000	1.000
Kindergarten	0.000	0.000	0.000	1.000
Primary school	0.000	0.000	0.000	0.999
Intermediate	0.000	0.000	0.000	1.000
Teeth brushing frequency (None per day)				
One time per day	0.000	0.000	0.000	1.000
two times per day	0.00	0.000	0.000	1.000
Three times or more per day	0.000	0.000	0.000	1.000
Flossing frequency (None per day)				
One time per day	0.000	0.000	0.000	1.000
Nearest primary health institution (Private)				
Government	1.678E+20	0.000	0.000	0.999
Last visit to the dental clinic in the previous 6 months (None)				
Once	0.000	0.000	0.000	0.999
Twice	0.000	0.000	0.000	1.000
More than 3 times	3.237E+104	0.000	0.000	0.999
Fathers’ education (None)				
Primary school	NA	NA	NA	NA
Intermediate school	0.000	0.000	0.000	1.000
Secondary	1.426E+12	0.000	0.000	1.000
Graduates	152222.289	0.000	0.000	1.000
Mothers’ education (Graduates)				
Primary school	0.858	0.000	0.000	1.000
Intermediate school	1.194E+48	0.000	0.000	1.000
Secondary	4.707E+17	0.000	0.000	0.999
Family income (21,000 – 40,000)				
≤10000	0.000	0.000	0.000	0.997
10000-20000	2.076E+116	0.000	0.000	1.000

## Discussion

This study investigated the prevalence of dental caries among children diagnosed with ASD in Arar, Northern Borders province, Saudi Arabia. Establishing baseline information on the dental health of children with ASD in Saudi Arabia is increasingly important. There is a notable lack of studies addressing the general health and specific dental health of autistic children. This study aimed to gather data on a group of autistic children in Arar city, focusing on caries occurrence with respect to sociodemographic characteristics. This information is intended to assist policymakers in creating effective oral health education programs tailored to children with ASD and similar disabilities.

The findings revealed a significant prevalence of caries among these children, with 61.4% exhibiting caries (DMFT > 0). This is similar to Jaber’s study based in the United Arab Emirates, where he noted the overall prevalence to be 77.0% [10]. The mean DMFT and deft for all the ASD children were 0.64 ± 1.33 and 0.91 ± 1.78, respectively. This level of dental caries is considered low according to the DMF index [21]. These findings are in agreement with other regional and international studies, which reported lower caries prevalence in children with ASD [22-25]. Age emerged as a significant predictor of caries prevalence in our study, with older children showing higher rates of caries. This finding is consistent with existing literature, suggesting that as children with ASD grow older, they may face cumulative challenges in maintaining adequate oral hygiene and accessing routine dental care [26]. The prevalence of decayed, missing, and filled teeth was seen to rise as individuals aged, largely due to the prolonged exposure of teeth to factors that promote decay, particularly affecting newly erupted second permanent molars during adolescence.

In our study, the parents' responses regarding the frequency of toothbrushing were noteworthy, particularly given the behavioral challenges and poor motor skills of children with ASD, which can make toothbrushing difficult for parents. However, a large number of individuals did not practice brushing at all. This contrasts with the generally positive attitude of Saudi parents of children with disabilities towards oral hygiene practices [22,23,27,28]. Generally, children with ASD often exhibit behavioral challenges that can significantly impact their oral health [29]. These challenges include difficulties in maintaining attention, communication deficits, and repetitive behaviors, all of which can interfere with establishing effective oral hygiene routines [30]. Behavioral challenges such as rigidity in routines, repetitive behaviors, and sensory-seeking behaviors also contribute to difficulties with oral hygiene among children with ASD [31]. For instance, insistence on sameness and resistance to changes in daily routines may disrupt regular toothbrushing schedules. Additionally, repetitive behaviors such as hand-flapping or rocking may interfere with the effective use of dental hygiene tools and techniques [30]. The large number of patients who had not visited the dentist in the past six months may be attributed to the perceived behavioral challenges as well as the reduced pain sensitivity and higher pain tolerance in children with ASD [9,24,25,32]. Similarly, a 2011 study found that over half of the study population had never been to a dental clinic, with the primary reason being the absence of dental pain or complaints [32].

Moreover, sensory sensitivities represent a hallmark feature of ASD and can profoundly influence oral health behaviors [33]. Many individuals with ASD have heightened sensitivity to tactile stimuli, taste, and texture, which can make brushing teeth with conventional toothbrushes and toothpaste challenging and uncomfortable. For example, the texture and flavor of toothpaste may be aversive to some children with ASD, leading to resistance or avoidance of toothbrushing altogether. Moreover, sensitivity to tactile sensations may result in discomfort when using dental floss or when undergoing dental examinations and treatments, thereby complicating oral hygiene practices. Research indicates that sensory sensitivities not only affect oral hygiene compliance but also impact the quality of dental care received by children with ASD [34]. Dental professionals may encounter challenges in conducting thorough examinations or providing treatments due to the child's aversion to sensory inputs associated with dental procedures. As a result, preventive measures such as fluoride applications or sealant placements may be compromised, further predisposing these individuals to dental caries and other oral health issues [35].

Communication deficits characteristic of ASD pose additional barriers to oral hygiene routines. Children with ASD may have difficulty understanding verbal instructions, expressing discomfort or pain, or communicating their oral health needs effectively [35]. This can impede their ability to cooperate during dental visits and hinder the establishment of consistent oral hygiene habits at home.

Understanding the complex interplay between behavioral challenges and sensory sensitivities is crucial for developing effective strategies to promote oral health in children with ASD. Dental providers can implement sensory adaptation techniques to create a more accommodating dental environment, such as using dimmed lights, minimizing noise levels, or providing weighted blankets to promote a sense of security during dental visits [36]. Furthermore, caregiver education plays a pivotal role in fostering positive oral health behaviors at home. Caregivers can learn strategies to desensitize their child to oral care routines gradually, such as introducing toothbrushing with a soft brush or flavored toothpaste in a familiar, calm setting [34]. Establishing predictable routines and incorporating visual supports can also enhance compliance with oral hygiene practices among children with ASD.

To foster better compliance with oral hygiene practices among children diagnosed with ASD, it is crucial to delve into the intricate dietary preferences and behaviors that significantly impact their oral health and predispose them to dental caries [37]. Understanding these dietary patterns is pivotal for developing scientifically grounded preventive strategies aimed at enhancing oral health outcomes in this particular cohort. A prominent challenge in the dietary habits of children with ASD is their notable preference for high-sugar foods and beverages [38]. Extensive research highlights that individuals with ASD often exhibit selective eating behaviors, showing a propensity for foods rich in sugars, carbohydrates, and processed ingredients. Studies have also shown that children with ASD exhibit strong aversions to bitter foods [39]. These dietary choices markedly elevate the risk of dental caries by promoting prolonged exposure to fermentable carbohydrates, which serve as substrates for acid-producing bacteria in the oral cavity, thereby exacerbating the formation of dental plaque and leading to enamel demineralization and cavity formation.

Furthermore, texture sensitivity adds another layer of complexity to the dietary habits of children with ASD. Many individuals in this population experience aversions to specific food textures or consistencies, leading to a restricted dietary repertoire. Typically, their diet tends to include soft and processed foods that are easier to chew and swallow but often contain higher levels of sugars and starches [39]. Furthermore, the limited dietary repertoire due to texture aversions restricts the availability of nutrient-dense foods that support oral health, such as fibrous fruits and vegetables that aid in saliva production and mechanical cleansing of teeth. Consequently, these dietary preferences contribute significantly to compromised oral health outcomes, including the development of dental caries and erosion [37]. In a cross-sectional study based in Dammam, Saudi Arabia, Kotha et al. examined the caries occurrence and dietary and hygiene habits of children with ASD and found a higher predilection for caries in children who consumed over two spoonfuls of sugar, and those who consumed sugar in between mealtimes [40]. Additionally, children with ASD generally have a tendency to retain food in the mouth rather than swallowing it, often due to impaired tongue coordination, which further heightens their susceptibility to dental caries.

Lastly, in contrast to some studies that have identified socio-demographic factors, such as family income and parental education, as influential determinants of oral health outcomes in children with ASD [41,42], our findings did not demonstrate significant associations between these variables and caries prevalence. This discrepancy may be attributed to the relatively small sample size of our study, which limits our statistical power to detect such associations effectively. Future research with larger sample sizes and diverse socio-economic contexts is warranted to further explore these relationships.

Strengths and limitations

This study has several notable strengths. First, it addresses a significant gap in the literature by focusing on the prevalence of dental caries among children with ASD in Saudi Arabia, a population that has not been extensively studied. The use of standardized diagnostic criteria for dental caries, following the WHO recommendations, ensures consistency and reliability in the data collection process [43]. The study also benefits from the rigorous training and calibration of examiners, which minimizes inter-examiner variability and enhances the accuracy of the oral health assessments. Furthermore, the comprehensive questionnaire, capturing a wide range of demographic and behavioral variables, provides a holistic view of the factors influencing dental health in this population.

However, the study is not without its limitations. The reliance on self-reported data from parents or guardians, particularly concerning oral hygiene practices and socioeconomic status, introduces the potential for reporting bias. The study also did not collect dietary history for the participating children with ASD, which is an important factor influencing dental caries. The cross-sectional design of the study limits the ability to infer causality between the observed variables and dental caries. Additionally, the study is confined to a single hospital in the Northern Borders province, which may limit the generalizability of the findings to other regions or settings within Saudi Arabia. The exclusion of uncooperative children or those with incomplete responses could also introduce selection bias, potentially skewing the results. Finally, while the study aims to cover a broad age range, the upper limit of 17 years may not capture the full spectrum of dental health issues that could be present in older adolescents or young adults with ASD. Despite these limitations, the study provides valuable insights and highlights the need for future research to address the identified gaps and further explore this important area.

## Conclusions

This study provides crucial insights into the prevalence and predictors of dental caries among children diagnosed with ASD in Arar, Saudi Arabia. Our findings highlight a significant caries prevalence of 61.4%, with older age emerging as a notable predictor. Despite notable challenges in oral hygiene practices among these children, no significant associations were found with socio-demographic factors. These findings highlight the urgent need for tailored oral health interventions and further research to mitigate dental health disparities in this vulnerable population.

## Appendices

**Table 7:** Autism questionnaire SAR: Saudi Arabian Riyal

Category	Variable	Options
General information	Age	—
	Gender	Female/Male
	Nationality	—
	Level of education	Nursery/Kindergarten/Primary school/Intermediate school/Secondary school/None
	Total number of siblings	—
	Birth order among siblings	—
	Diagnosed with autism	Yes/No
Oral hygiene behaviors	Teeth brushing frequency	None per day/Once per day/Twice per day/Three times or more per day
	Flossing frequency	None per day/Once per day/Twice per day/Three times or more per day
	Nearest primary health institution	Governmental/Private
	Last dental clinic visit (past 6 months)	None/Once/Twice/More than 3 times
Parents/guardians information	Father’s level of education	None/Primary school/Intermediate school/Secondary school/Graduate studies
	Mother’s level of education	None/Primary school/Intermediate school/Secondary school/Graduate studies
	Average family income per month	≤10,000 SAR/10,000–20,000 SAR/20,000–40,000 SAR/>40,000 SAR
